# Supplementary material for: Isolation of Two New Compounds and Other Constituents from Leaves of Piper crocatum and Study of Their Soluble Epoxide Hydrolase Activities
Source: Molecules. 2019 Jan 30;24(3):489. doi: 10.3390/molecules24030489 (PMC6384562; doi:10.3390/molecules24030489)
Supplement: Supplementary file 1 [file molecules-24-00489-s001.pdf]

Supplementary Data

Communication

Isolation of Two New Compounds and Other Constituents from Leaves of *Piper crocatum* and Study of Their Soluble Epoxide Hydrolase Activities

Hong Xu Li [1], Seo Young Yang [1,*], Young Ho Kim [1,*] and Wei Li [2,*]

[1] College of Pharmacy, Chungnam National University, Daejeon 34134, Korea ; charon0077@gmail.com
[2] Korean Medicine (KM) Application Center, Korea Institute of Oriental Medicine, Daegu 41062, Korea
* Correspondence: syyang@cnu.ac.kr (S.Y.Y.); yhk@cnu.ac.kr (Y.H.K.); liwei1986@kiom.re.kr (W.L.);
Tel.: +82-42-821-7321 (S.Y.Y.); +82-42-821-5933 (Y.H.K.); +82-53-940-3874 (W.L.)

Received: 16 January 2019; Accepted: date; Published: date

Figure S1. HR-ESI-MS spectrum of compound **1** ---------- 4

Figure S2. ^{1}H NMR spectrum of compound **1** in methanol-d_4 (600 MHz) ---------- 5

Figure S3. ^{13}C NMR spectrum of compound **1** in methanol-d_4 (100 MHz) ---------- 6

Figure S4. HMQC spectrum of compound **1** ---------- 8

Figure S5. HMBC spectrum of compound **1** ---------- 10

Figure S6. COSY spectrum of compound **1** ---------- 11

Figure S7. ROESY spectrum of compound **1** ---------- 12

Figure S8. HR-ESI-MS spectrum of compound **2** ---------- 14

Figure S9. ^{1}H NMR spectrum of compound **2** in methanol-d_4 (600 MHz) ---------- 15

Figure S10. ^{13}C NMR spectrum of compound **2** in methanol-d_4 (100 MHz) ---------- 16

Figure S11. HMQC spectrum of compound **2**--17
Figure S12. HMBC spectrum of compound **2**--18
Figure S13. COSY spectrum of compound **2**--18
Figure S14. ROESY spectrum of compound **2**---19

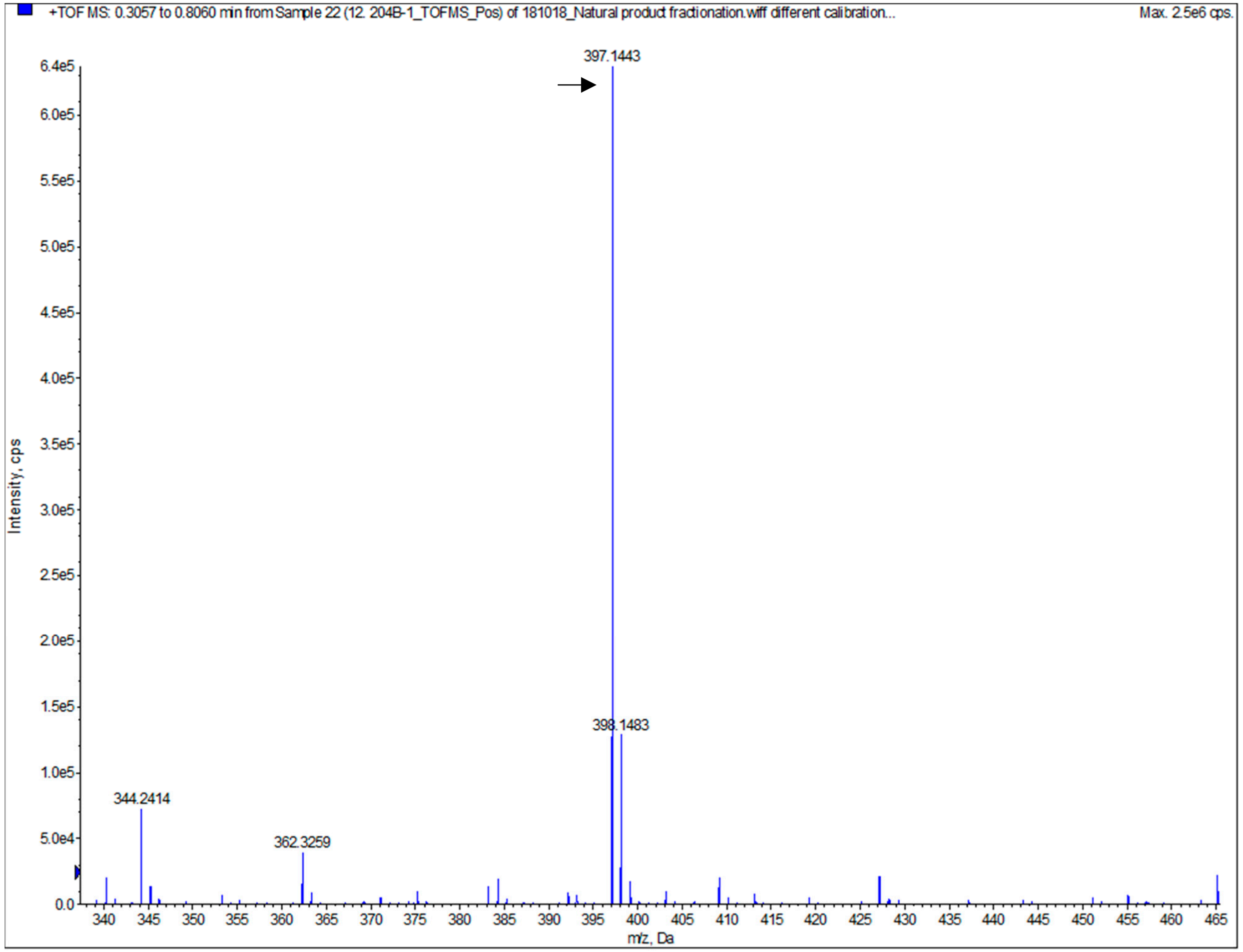
+TOF MS: 0.3057 to 0.8060 min from Sample 22 (12. 204B-1_TOFMS_Pos) of 181018_Natural product fractionation.wiff different calibration...
Max. 2.5e6 cps.
Intensity, cps
m/z, Da
397.1443
398.1483
344.2414
362.3259

Figure S1. HR-ESI-MS spectrum of compound **1**.

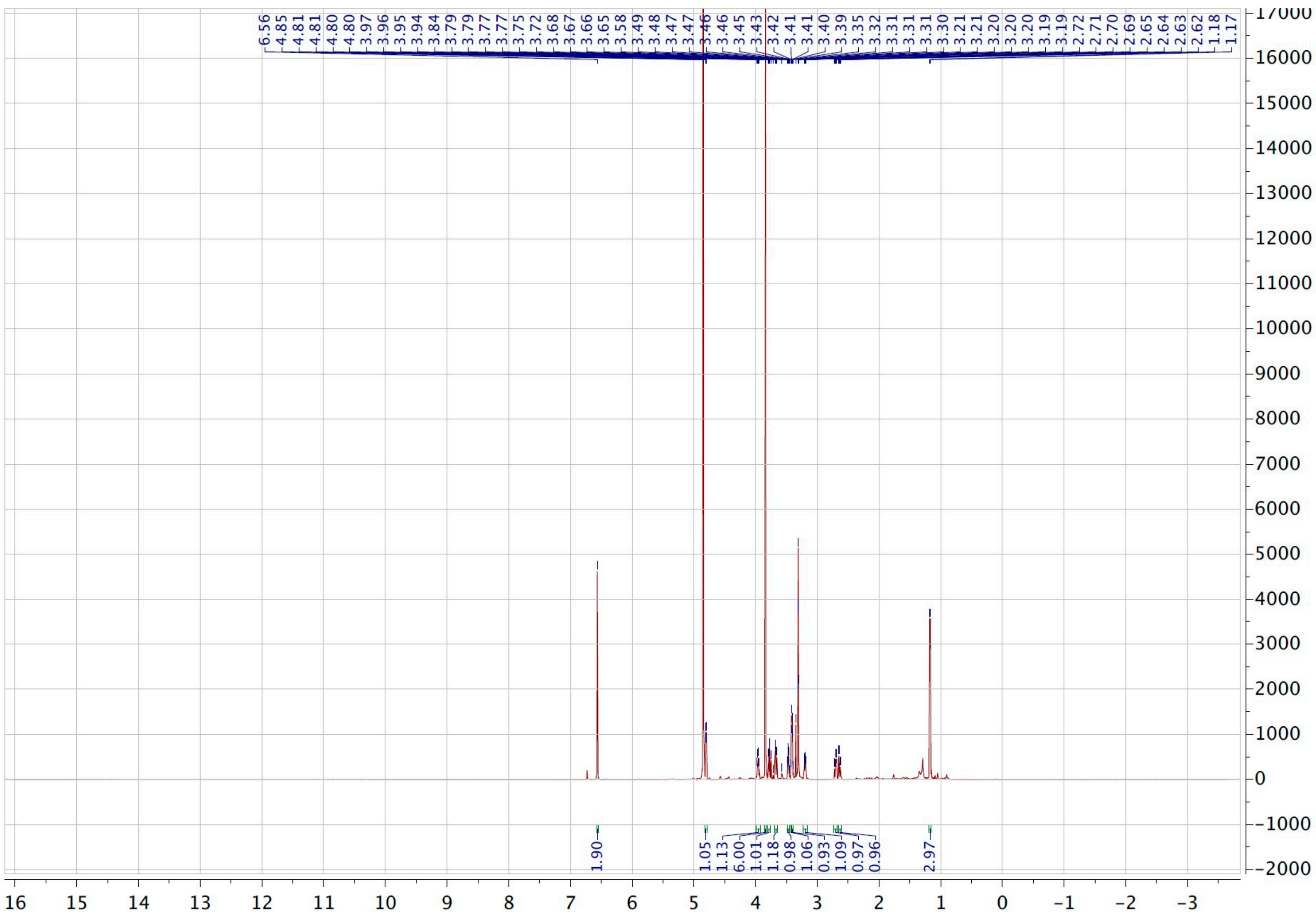

Figure S2. ^{1}H NMR spectrum of compound **1** in methanol-d_4 (600 MHz).

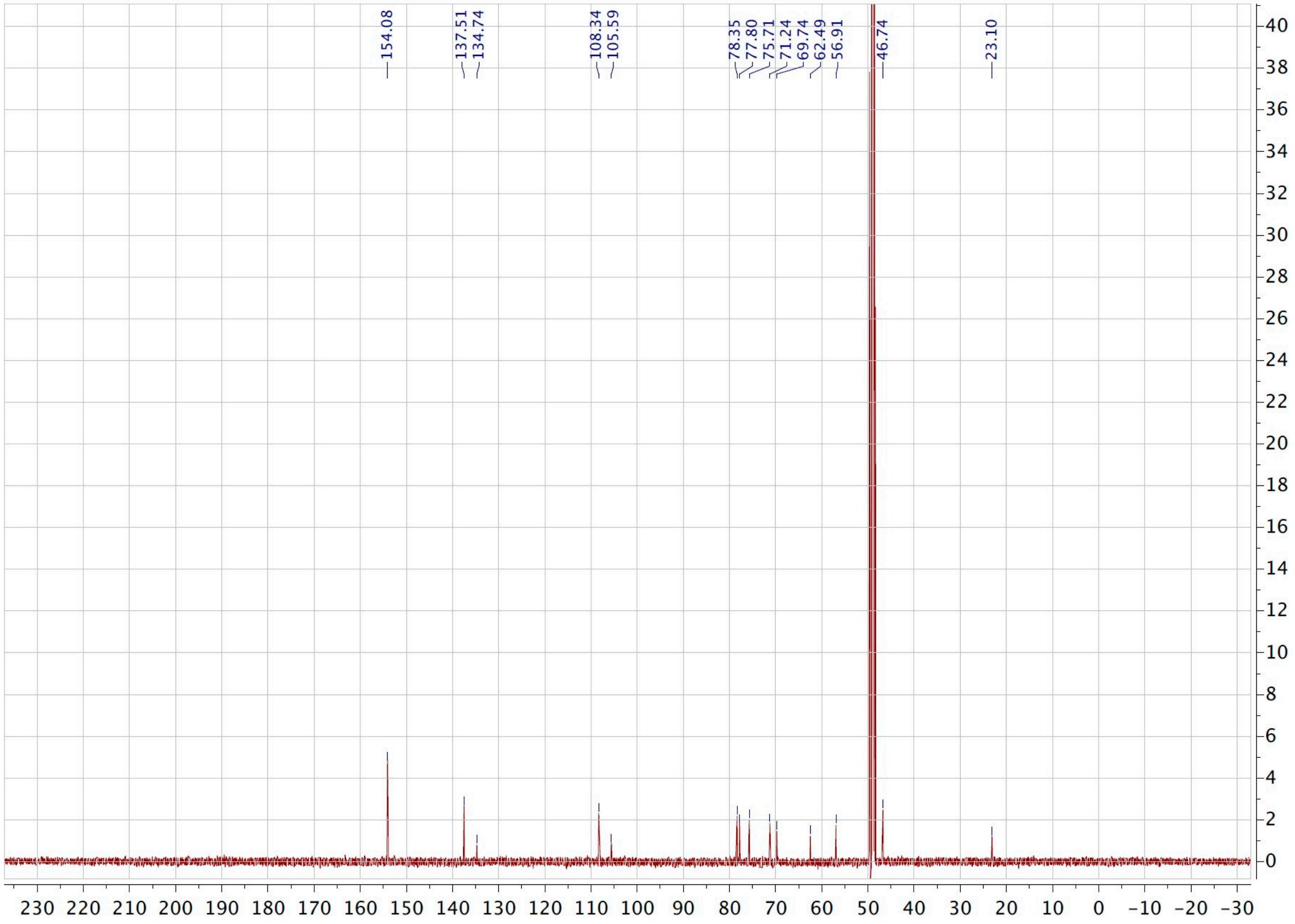

Figure S3. ^{13}C NMR spectrum of compound **1** in methanol-d_4 (150 MHz).

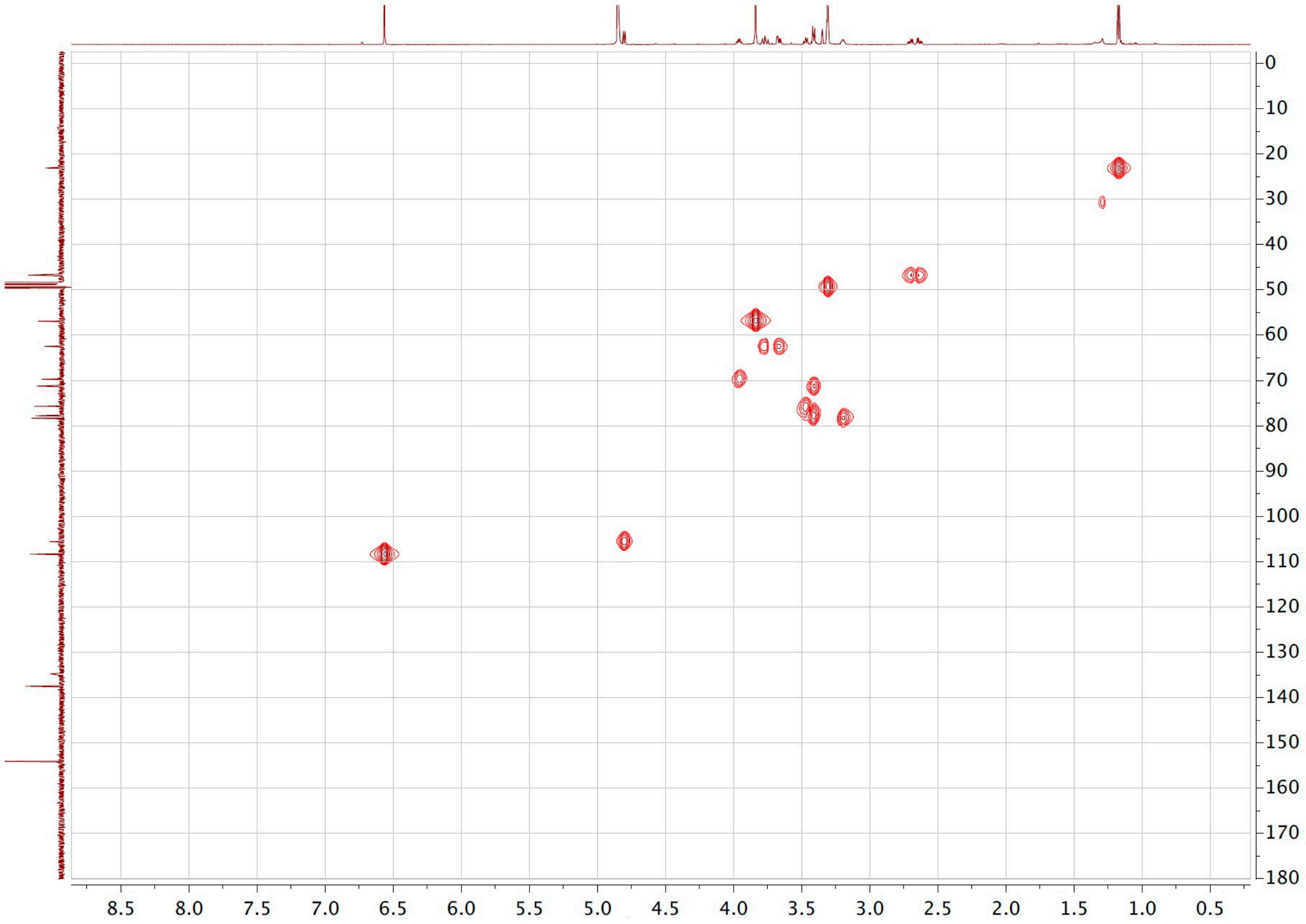

8.5
8.0
7.5
7.0
6.5
6.0
5.5
5.0
4.5
4.0
3.5
3.0
2.5
2.0
1.5
1.0
0.5
0
10
20
30
40
50
60
70
80
90
100
110
120
130
140
150
160
170
180

1

Figure S4. HMQC spectrum of compound.

1

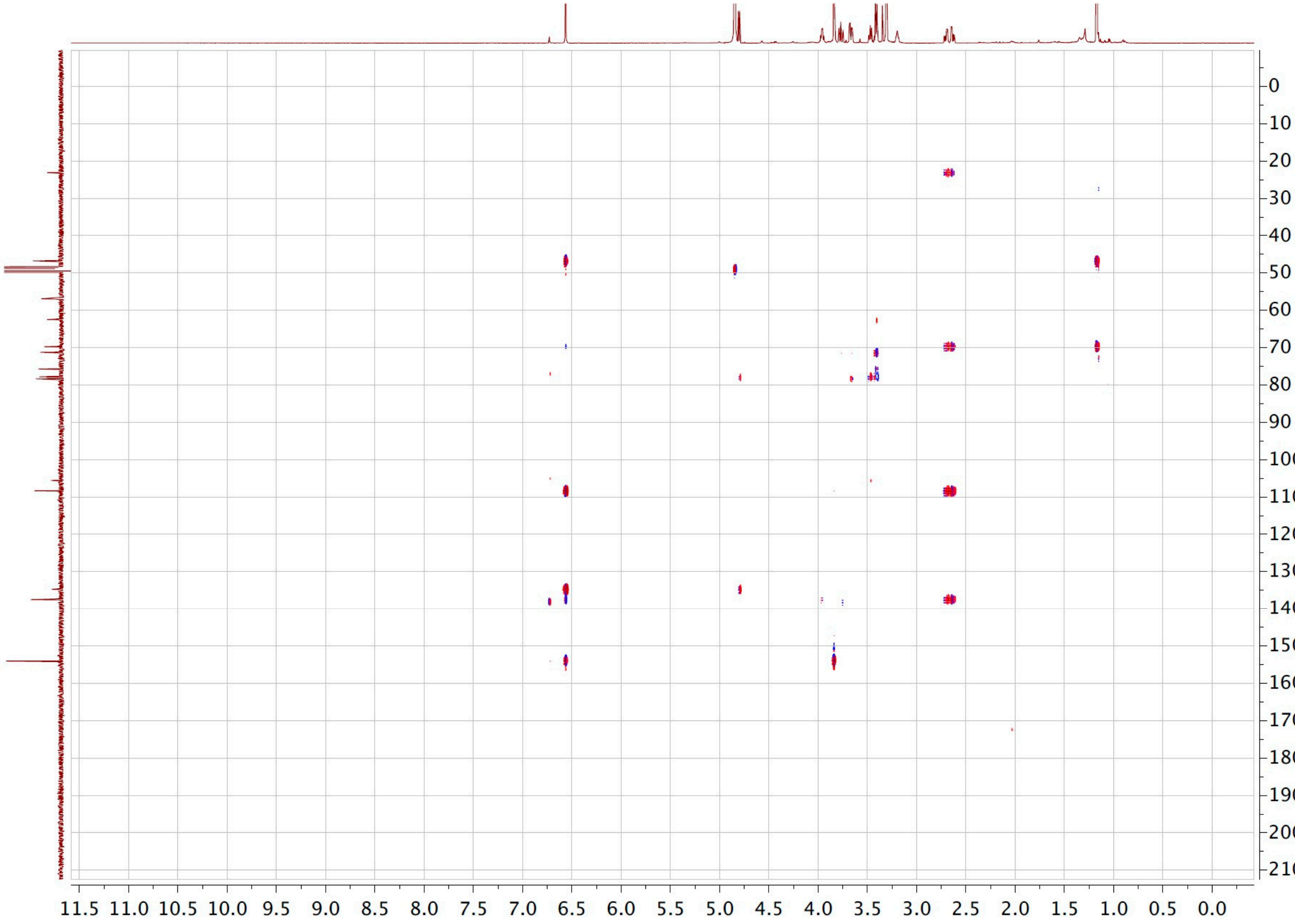
0
10
20
30
40
50
60
70
80
90
100
110
120
130
140
150
160
170
180
190
200
210
11.5 11.0 10.5 10.0 9.5 9.0 8.5 8.0 7.5 7.0 6.5 6.0 5.5 5.0 4.5 4.0 3.5 3.0 2.5 2.0 1.5 1.0 0.5 0.0

1

Figure S5. HMBC spectrum of compound.

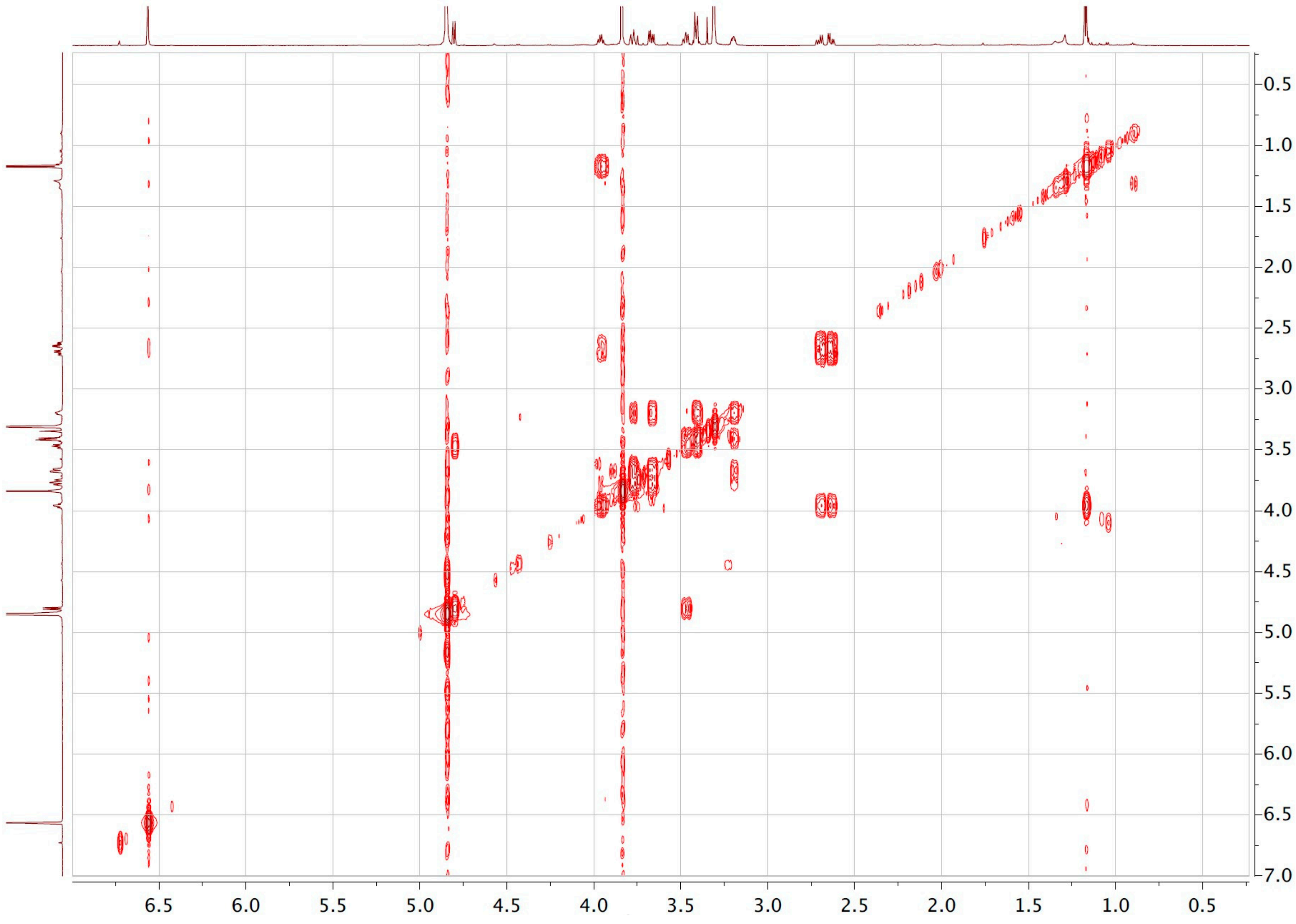

1

Figure S6. COSY spectrum of compound.

1

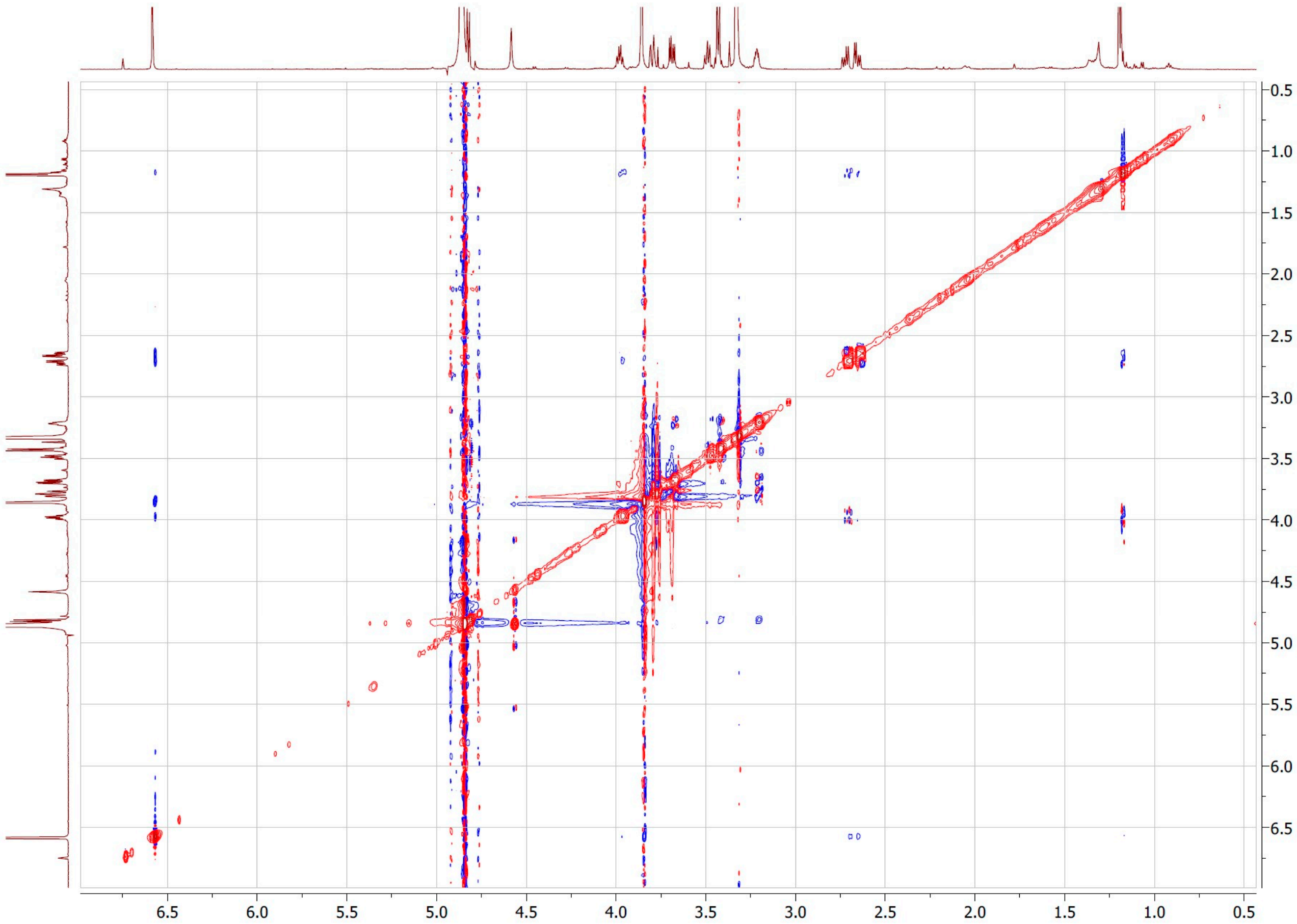

Figure S7. ROESY spectrum of compound **1**

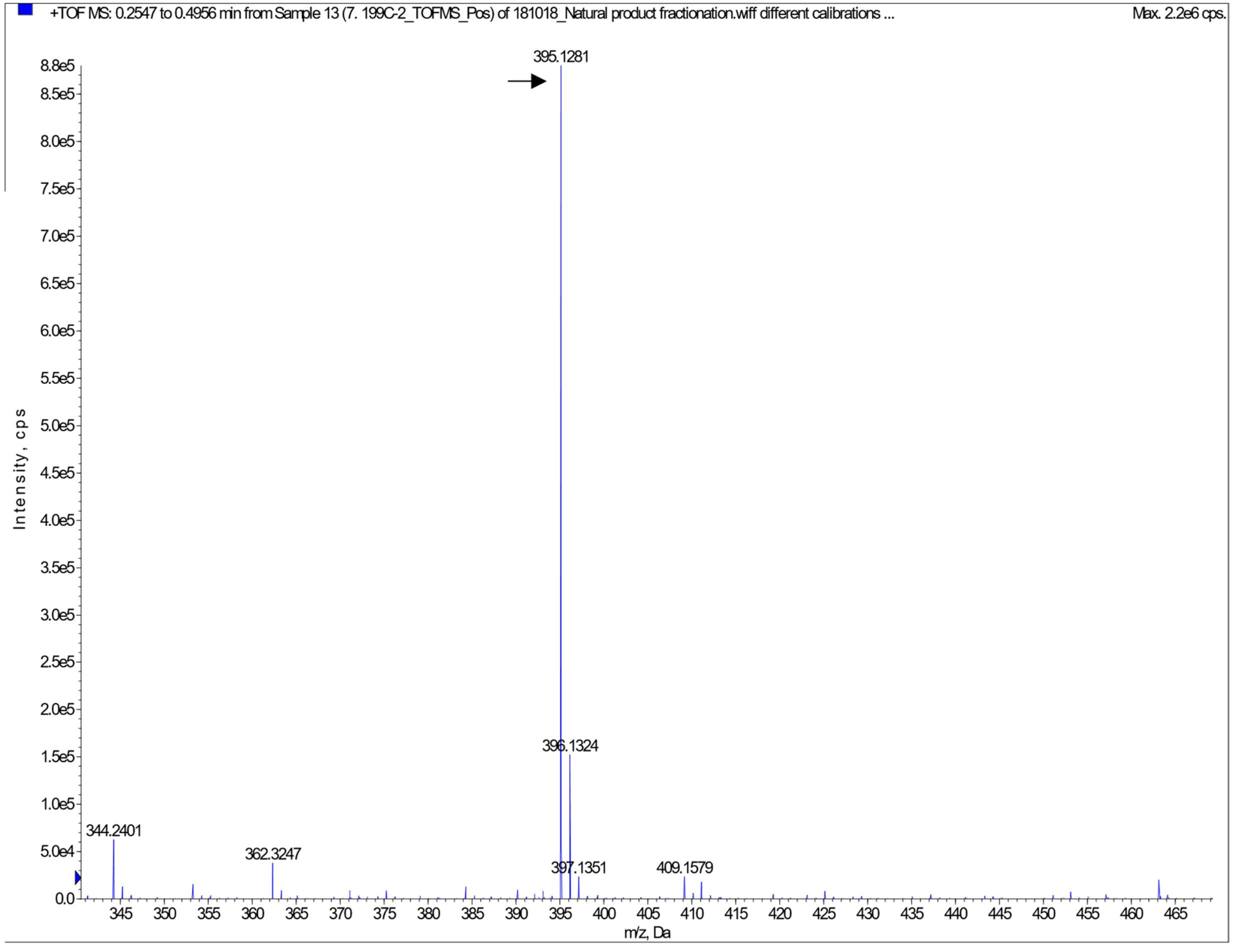
+TOF MS: 0.2547 to 0.4956 min from Sample 13 (7. 199C-2_TOFMS_Pos) of 181018_Natural product fractionation.wiff different calibrations ...
Max. 2.2e6 cps.
395.1281
396.1324
344.2401
362.3247
397.1351
409.1579
Intensity, cps
m/z, Da

Figure S8. HR-ESI-MS spectrum of compound **2**.

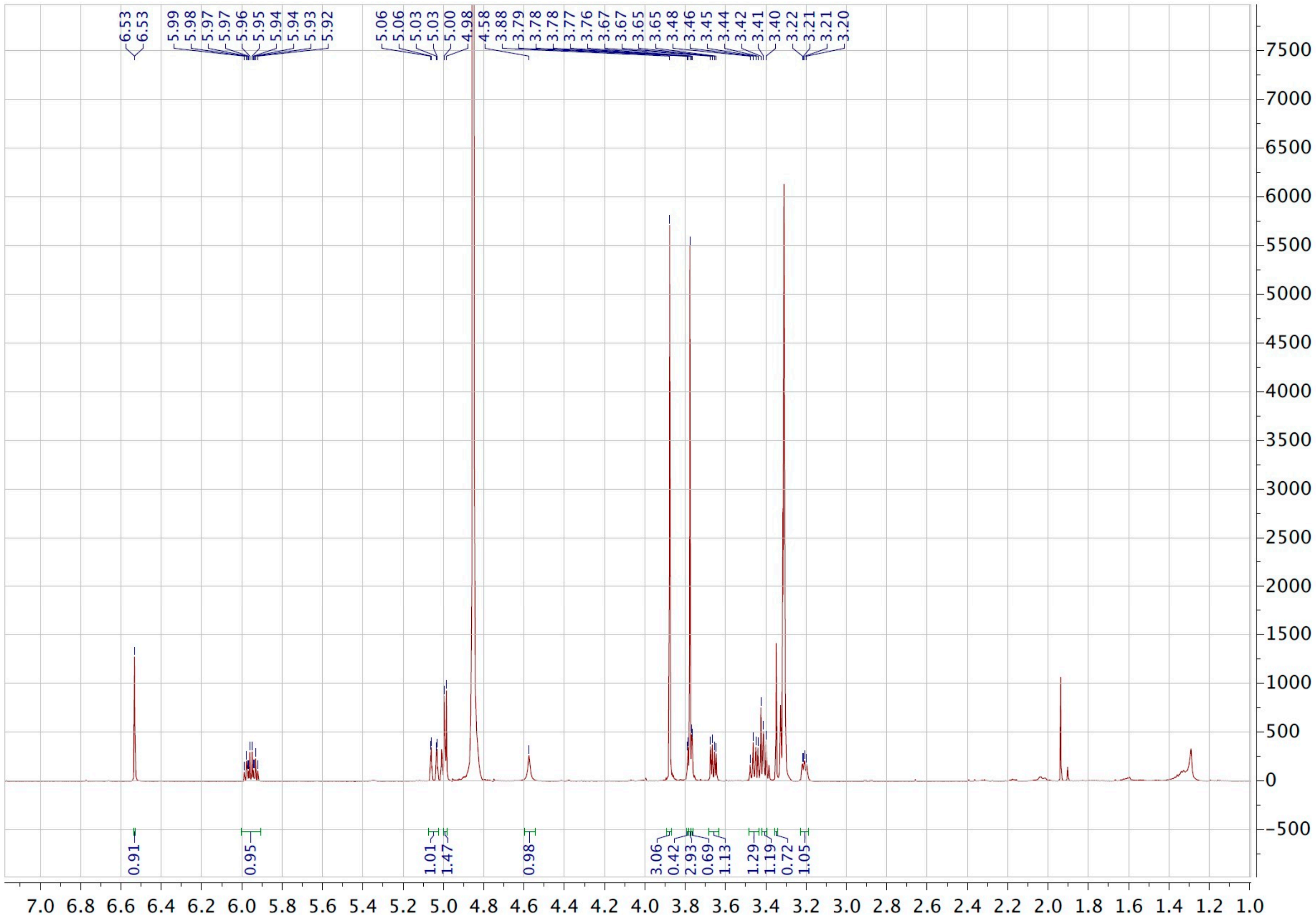

Figure S9. ^{1}H NMR spectrum of compound **2** in methanol-*d*$_4$ (600 MHz).

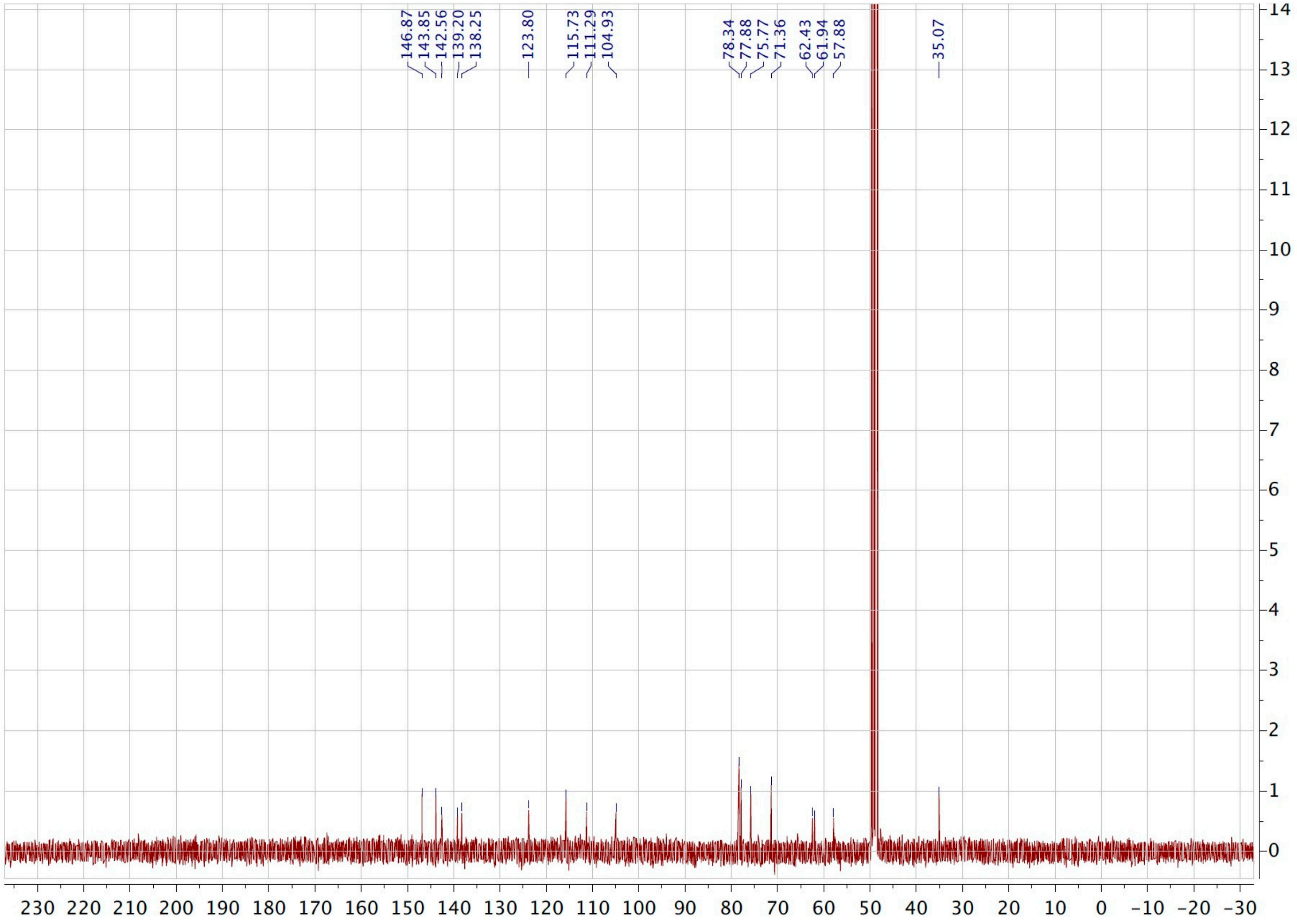

Figure S10. ^{13}C NMR spectrum of compound **2** in methanol-*d₄* (150 MHz).

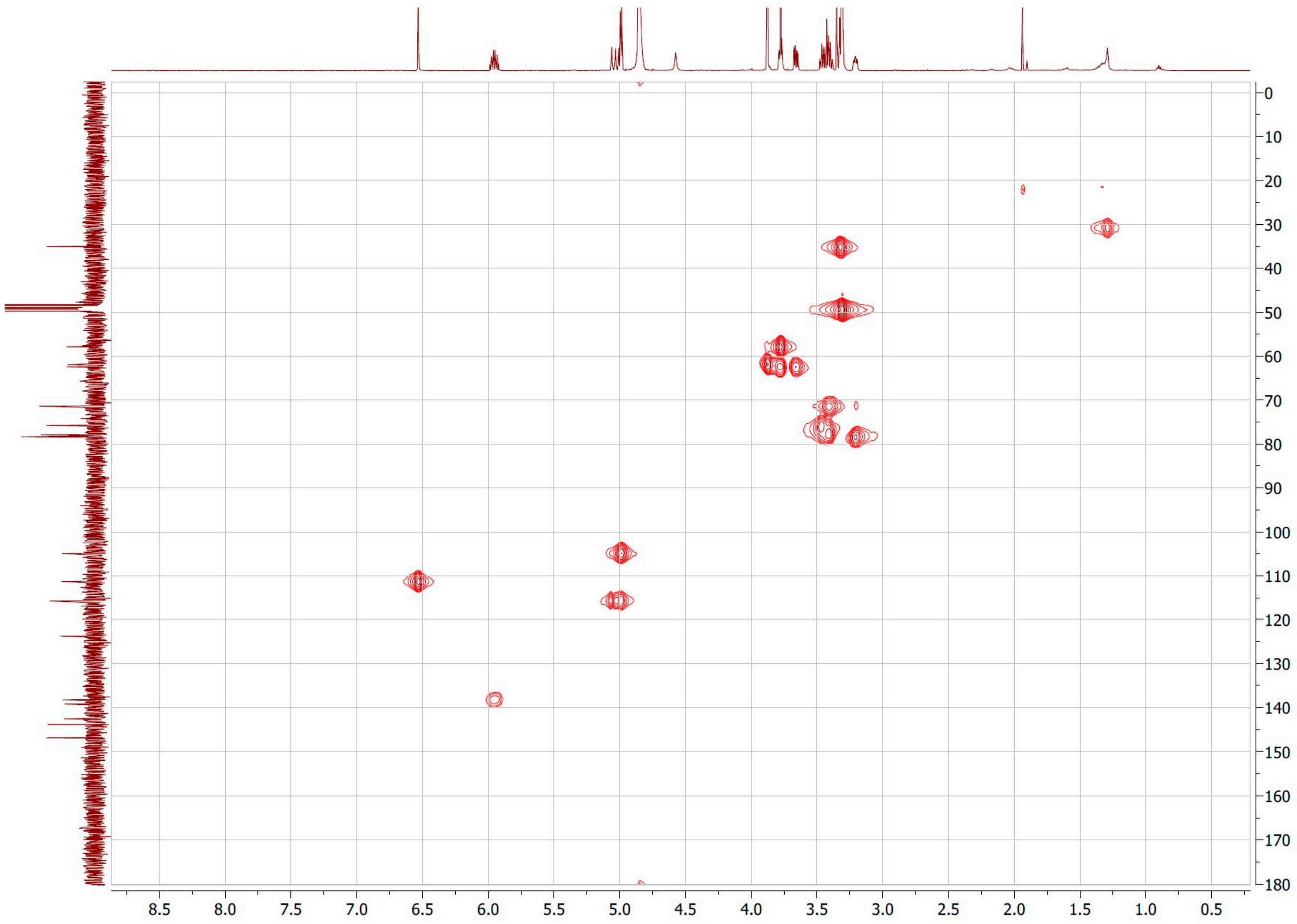

Figure S11. HMQC spectrum of compound **2**.

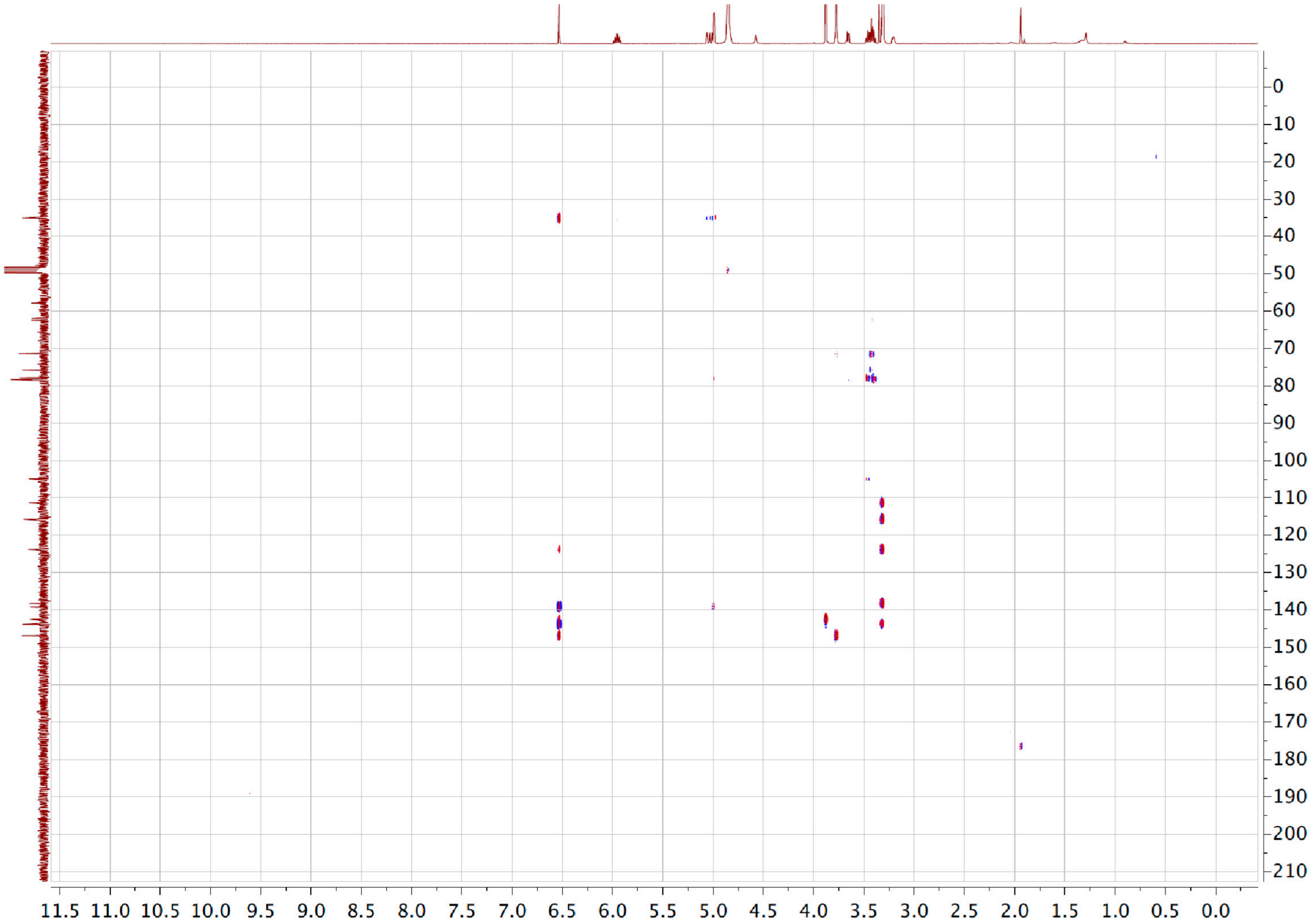

Figure S12. HMBC spectrum of compound **2**.

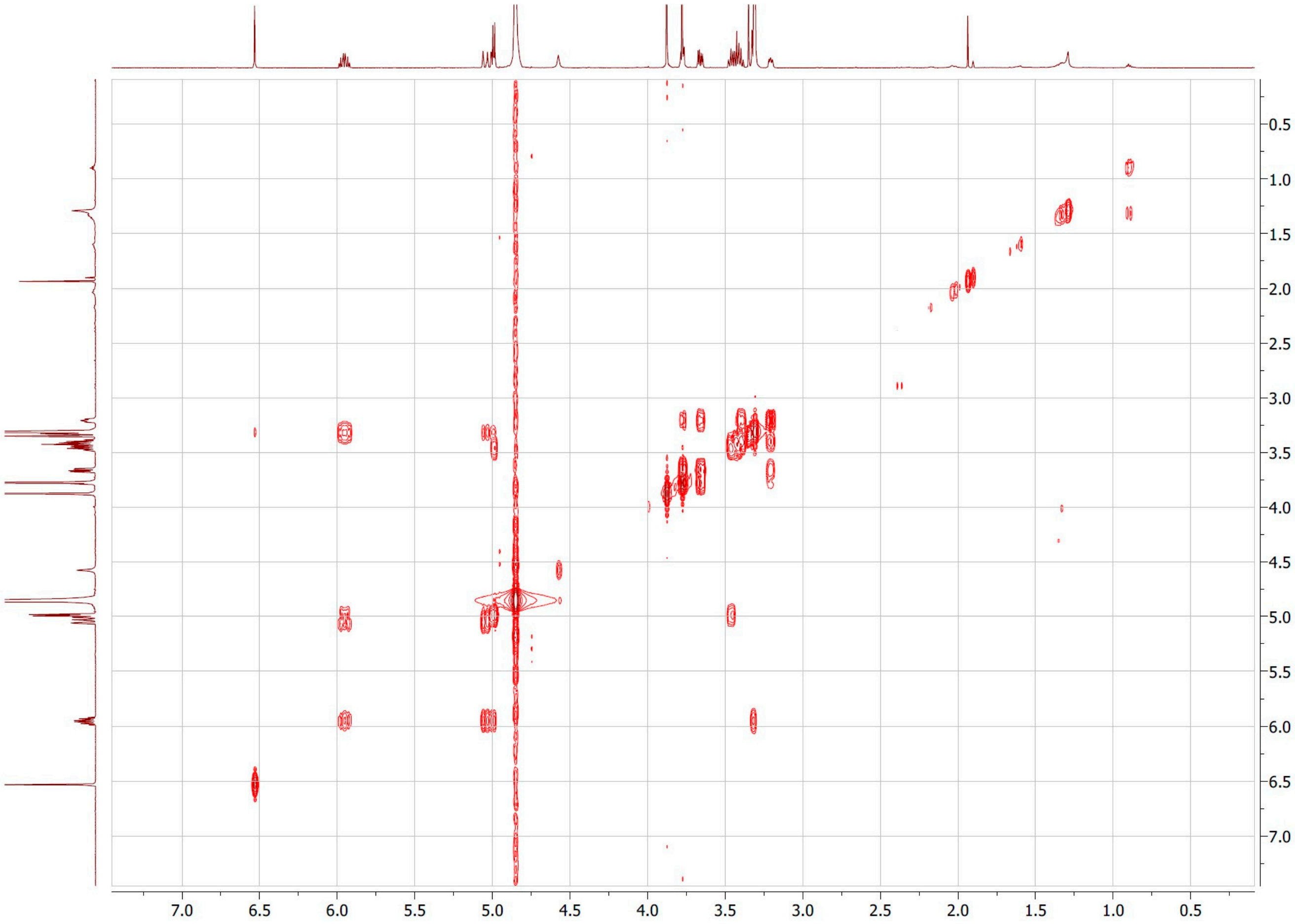

Figure S13. COSY spectrum of compound **2**.

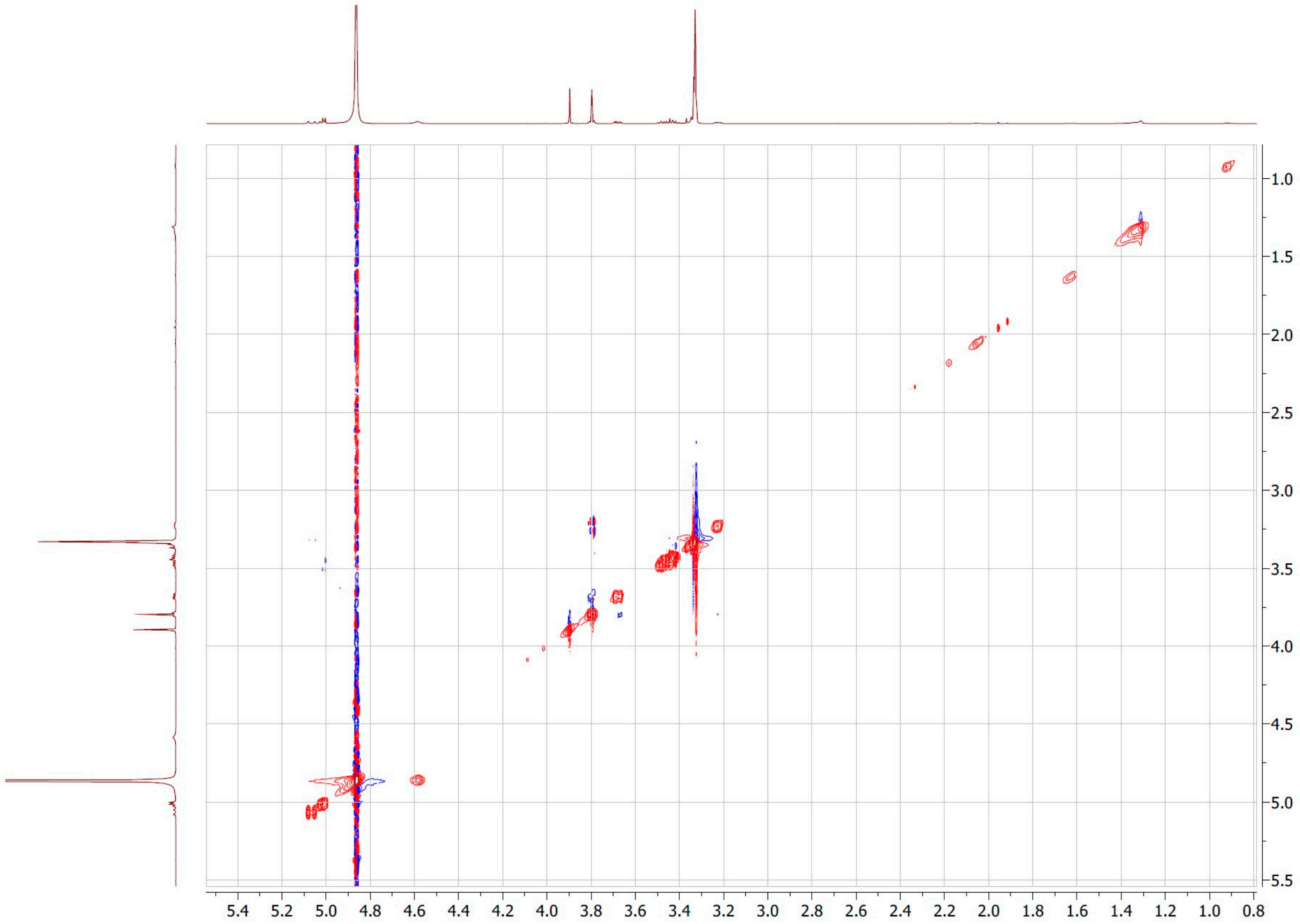

Figure S14. ROESY spectrum of compound **2**.
